# Saccadic landing positions reveal that eye movements are affected by distractor-based retrieval

**DOI:** 10.3758/s13414-022-02538-8

**Published:** 2022-08-17

**Authors:** Lars-Michael Schöpper, Markus Lappe, Christian Frings

**Affiliations:** 1 Department of Cognitive Psychology, University of Trier, Trier, Germany; 2 Department of Psychology, University of Muenster, Muenster, Germany

**Keywords:** Attention, Perception, Eye movements, S-R binding, Output-related saccades

## Abstract

Binding theories assume that stimulus and response features are integrated into short-lasting episodes and that upon repetition of any feature the whole episode is retrieved, thereby affecting performance. Such binding theories are nowadays the standard explanation for a wide range of action control tasks and aim to explain all simple actions, without making assumptions of effector specificity. Yet, it is unclear if eye movements are affected by integration and retrieval in the same way as manual responses. We asked participants to discriminate letters framed by irrelevant shapes. In Experiment 1, participants gave their responses with eye movements. Saccade landing positions showed a spatial error pattern consistent with predictions of binding theories. Saccadic latencies were not affected. In Experiment 2 with an increased interval between prime and probe, the error pattern diminished, again congruent with predictions of binding theories presuming quickly decaying retrieval effects. Experiment 3 used the same task as in Experiment 1, but participants executed their responses with manual key presses; again, we found a binding pattern in response accuracy. We conclude that eye movements and manual responses are affected by the same integration and retrieval processes, supporting the *tacit* assumption of binding theories to apply to any effector.

## Introduction

Throughout the day we interact with our environment with quite simple movements. Whether postponing the morning alarm by pressing the snooze button, grabbing a cup of coffee to take a sip, or turning the door handle to leave the house – all of these movements are considered “actions.” In the tradition of ideomotor theory (see Shin et al., 2010, and Stock & Stock, 2004, for reviews), modern action control theories define actions as being intentional movements that are performed with an anticipated goal in mind (Frings et al., 2020; Prinz, 1998). In the above examples, the action execution is performed by the manual system while the visual system is involved to gather stimulus information. However, the execution of actions is not limited to the manual system; swinging a leg, moving the tongue, or looking somewhere can be defined as an action, too, when such movements are done intentionally with an anticipatory goal about environmental consequences in mind. In other words, action control theories are not effector-specific.

One theory that aims to describe the processes involved in performing an action is the theory of event coding (Hommel, 1998, 2004; Hommel et al., 2001): When responding to a stimulus, the stimulus, its features (even if completely task-irrelevant, Frings et al., 2007), and the response are integrated into a short episodic memory trace, also known as an *event file*. The theory proposes that, when any information of the event file repeats in the subsequent action, the whole event file is retrieved. This mandatory retrieval affects performance depending on whether the aspects of the current action match or deviate from the previous event file.

The processes of integration and retrieval (Frings et al., 2020; Laub et al., 2018) are assumed to underlie all intentional actions (e.g., Frings et al., 2020; Hommel, 2004). Ultimately, these processes are thought to be active in many experimental paradigms that involve a sequential design (see Frings et al., 2020). Examples include priming (Henson et al., 2014), task switching (e.g., Koch et al., 2018), and conflict tasks (Davelaar & Stevens, 2009; Gratton et al., 1992). Moreover, given the rather broad definition of what constitutes an action, binding processes are implicitly assumed to be effector-invariant. Congruent with that, when the effector (hand or foot) giving the response changes, binding effects can still be observed, suggesting event-file representation being rather abstract than effector-specific (e.g., Moeller et al., 2015).

Effector-invariance of binding theories predicts that binding effects should be observed irrespective of whether the response is executed with, for example, a manual or an eye movement. Common action control processes for all effectors also suggests similar neurophysiological processing during action planning until response execution. In a number of binding studies (e.g., Frings et al., 2007; Laub et al., 2018), the visual modality is used to gather input, while the manual modality gives the response, suggesting the activation and interaction of visual and motor areas (e.g., Pollmann et al., 2006). However, in eye-movement control, the eye (movement) both gathers information and gives the response (see, e.g., Huestegge, 2011, for an output-related view of eye movements in multitasking), interlinking brain areas responsible for processing visual input and for saccade generation – and makes the generation of manual movements unnecessary.

The oculomotor system is largely distinct from the motor system for limb movements. It involves, mainly, the superior colliculus (Wurtz & Goldberg, 1972), the frontal eye field (Bruce & Goldberg, 1985; Schall, 2015), and the lateral intraparietal area (Bisley & Goldberg, 2010). This specificity might support effector specificity of binding. On the other hand, planning a visually guided eye movement involves processes such as visual localization, identification, attention, etc., that might be shared across effectors. While some studies suggest effector-specific processing of visual and motor planning in the brain (e.g., Gallivan et al., 2019), others show that saccades are modulated by brain areas (e.g., the dorsolateral prefrontal cortex; e.g., Meeter et al., 2010; Trottier & Pratt, 2005) that are thought to play an important role in goal-directed cognitive control (e.g., Miller & Cohen, 2001). Additionally, areas like the posterior parietal cortex (e.g., Andersen & Buneo, 2002; Fattori et al., 2017), the right insula (Ho et al., 2009), or the superior colliculus (Werner et al., 1997) affect the planning and/ or execution of both eye and arm movements.

Thus, the neurophysiological evidence neither rules out nor confirms the possibility of eye movements being affected by retrieval-based binding processes. However, given some rather distinct pathways for saccade generation opens up the possibility of effector-specificity – which might be the reason for differences that have been previously observed between manual and eye responses (e.g., for inhibition-related effects, Ding et al., 2016; Eng et al., 2017; Malienko et al., 2018; Taylor & Klein, 2000; in Hick’s law, Kveraga et al., 2002; Lawrence et al., 2008; see Proctor & Schneider, 2018, for a review; localization responses to moving targets, Lisi & Cavanagh, 2017; and several other tasks, see Bompas et al., 2017). Moreover, the joint Simon effect (Sebanz et al., 2003) is not observed for saccadic responses (Liepelt et al., 2019), thus raising doubt that all actions are processed in the same way irrespective of the effector involved.

Recently, Hilchey, Rajsic, et al. (2018) conducted a study in which they functionally differentiated effectors by task demands: In each trial, participants performed two tasks, one involving a saccade, the other a manual action, during the same experimental sequence. Different target identities^1^ (i.e., “x” or “+”) appeared left or right from a fixation cross. Target identity and location were systematically varied to fully or partially repeat, or fully change. Participants had to make saccades to every appearing target and then discriminate each target identity with a keypress. The manual responses revealed a binding effect indicated by an advantage of full repetitions over partial repetitions (and a benefit of full changes where nothing got retrieved). The saccadic responses revealed a benefit for location changes, that is, inhibition of return (IOR; Klein, 2000; Maylor & Hockey, 1985) without any influence of repeating or changing target identity. The authors argue that the eye movements in their experimental design were used to orient to a target (i.e., to localize it) without processing the target’s identity while the manual responses had to discriminate target identity.

The absence of a binding pattern in tasks where target identity discrimination is unnecessary is commonly observed in detection and localization (see Huffman et al., 2018; Schöpper, Hilchey, et al., 2020) performance (when responding to visual stimuli; Schöpper & Frings, 2022). This suggests that target identity discrimination is crucial for observing binding effects (see also Hilchey, Leber, & Pratt, 2018; Hilchey, Rajsic, et al., 2018; Huffman et al., 2020). Moreover, partial repetition costs in detection and localization performance can be observed if the location of the target has to be further processed after identifying it to give a response (Schöpper et al., 2022; Hilchey et al., 2020). This suggests that the processing of a general post-selection stage prior to responding produces a binding pattern. Therefore, the data pattern of Hilchey, Rajsic, et al. (2018) – that is, binding affecting manual but not saccadic responses – might have arisen from task demands rather than effector specificity. Accordingly, the effector-invariance in action control (e.g., Frings et al., 2020; Hommel, 2004; Prinz, 1998) predicts that a target-identity discrimination task executed with eye movements should yield a binding pattern. In turn, similar processes until response generation (Schöpper et al., 2022) should apply irrespective of the effector executing the response.

## Current study

In the current study we investigated whether eye movements are affected by retrieval in the same way as manual movements are. To do so, we asked participants to discriminate target letters by executing a response with eye movements in Experiment 1, discriminate target letters with eye movements with an increased response-stimulus-interval (RSI) in Experiment 2, and with manual movements in Experiment 3. Crucially, the target letters were framed by a repeating or non-repeating shape, which was irrelevant for task execution. If eye movements are affected by integration and retrieval processes, the shape should be integrated with the response and upon repetition cause retrieval. Thus, we expect a benefit if response and irrelevant shape fully repeat and an interference if repetition is only partial (e.g., Frings et al., 2007). Full changes of response and irrelevant shape should produce no interference because nothing is retrieved (e.g., Frings et al., 2020; Hommel, 2004). Such binding effects typically manifest themselves in a crossed data pattern (see, e.g., Hommel, 2004; discrimination tasks in Schöpper, Hilchey, et al., 2020). In the current study we expect responses to be fast and accurate if response and distractor fully repeat and fully change, but slower and less accurate if response and distractor only partially repeat. Note, comparable to effects with manual responses, we expected in Experiment 1 with a short RSI this “typical” interaction of stimulus x responses feature repetition. Yet, in Experiment 2 with a long RSI this pattern should be diminished (Frings, 2011; Hommel & Frings, 2020; Pastötter et al., 2021) as distractor-based retrieval decays quickly over time.

Experiment 3 used an identical design to Experiment 1 with the only difference being the effector with which the response was executed. This allows us to directly test the implicitly assumed effector invariance in binding theories.

## Experiment 1 (eye responses; short RSI)

### Participants

Twenty-seven university students and staff of the University of Münster participated for course credit, a monetary reward (4 €), or voluntarily. This sample size was based on Frings et al. (2007) and should, assuming a medium to high effect size of *d* = 0.6 and an error probability of α = 0.05 (one-tailed), yield a power of 1−β = 0.92 (G*Power, Version 3.1.9.2; Faul et al., 2007). All participants gave written informed consent. One participant reported a minor uncorrected refractive error that did not hinder task execution; all other participants reported normal or corrected-to-normal vision. One participant reported having problems looking at the lower target position. This was confirmed by the distribution of eye-movement data since the mean saccadic landing positions were heavily biased in the direction of the upper target position. The participant was excluded from analysis. Another participant reported having participated in an eye-tracking experiment investigating saccadic adaptation processes shortly before the testing session. To avoid distortion in response accuracy due to previously learned adaptation (e.g., Lappe, 2009), testing was aborted after the practice block and a full testing session took place a few days later. Although this participant had in total slightly more trials due to the repetition of the practice block, we decided to include the data, because they only had a minor numerical influence and thus did not affect the interpretation of the results. This resulted in a total sample size of 26 participants (17 women, nine men, *M*_*age*_ = 24.46 years, *SD*_*age*_ = 4.32, age range: 18–35).

### Apparatus and materials

Eye movements were recorded with an Eyelink 1000 system (SR Research, Ontario, Canada) with a frequency of 1,000 Hz. We always recorded the right eye. Stimuli were displayed on a screen with a display resolution of 1,152 × 870 pixels (px; approximately 35.80° × 26.91° of visual angle) and a refresh rate of 75 Hertz. A chin rest was positioned at approximately 62.7 cm in front of the screen. Testing took place in a dark room, dimly lit by indirect light. The fixation dot and two target dots, all with a diameter of 20 px (0.64° of visual angle), were presented on a grey background. The fixation dot was black and was presented at the left half of the screen (x-axis: 200 px or approx. 6.38° of visual angle left from center; y-axis: central). The two target dots were white and were presented at the right half of the screen (x-axis: 200 px or approx. 6.38° of visual angle right from the center), one 150 px above the other 150 px below the horizontal midline (300 px or approx. 9.57° of visual angle apart). All three dots (fixation and two target dots) were visible through a whole prime (until after response) or probe (until after response) presentation. After the participant established fixation at the fixation dot, the fixation dot was replaced by an instruction letter at the same position. Instruction letters were white and could be “P,” “R,” “B,” or “D,” and measured approximately 0.46° × 0.64° of visual angle (length x height). Instruction letters were framed by a distractor shaped like either a square or a circle with a side length or diameter of 60 px (1.83° of visual angle), respectively. The distractor consisted of a white contour of the shape (i.e., the shape was unfilled, therefore grey as the background).

### Design

The experiment used a 2 (response relation: repeated vs. changed) × 2 (distractor relation: repeated vs. changed) design. All variables were varied within subjects. Binding effects were computed as the interaction of response relation x distractor relation.

### Procedure

**Fig. 1:**
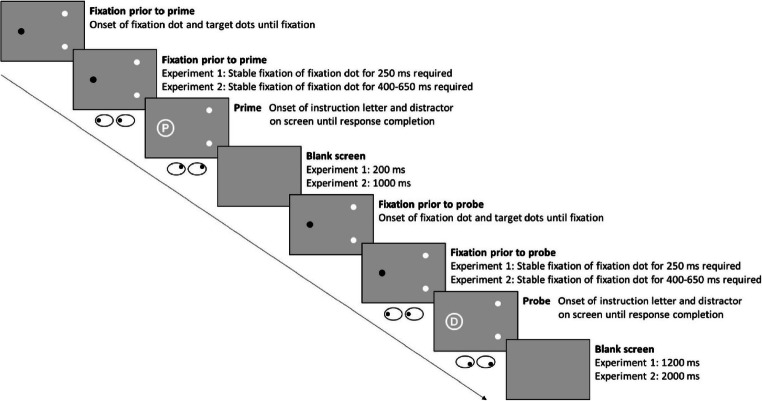
A prime-probe sequence as used in Experiment 1 and Experiment 2 (not drawn to scale). This example depicts a trial in which the response changes but the distractor repeats

A sequence was started by the simultaneous presentation of a black fixation dot and two white target dots. After a required stable fixation of the fixation dot for an interval of 250 ms, the prime started by an instruction letter framed by a distractor appearing at the position of the fixation dot. Eye position was monitored online. If the eye position deviated from the fixation point by more than 100 px (approx. 3.19°) during the fixation period the participant was requested to re-fixate and the fixation period started anew. The time between onset of the fixation dot and target letter onset was logged for all prime and probe fixations. Participants were instructed to look at the upper or lower dot on the right half of the screen, depending on the corresponding letter: The letters “P” and “R” indicated looking at the upper target dot, the letters “B” and “D” indicated looking at the lower target dot. After the participant looked at the corresponding target dot and gaze was within 100 px (approx. 3.19°) from the target for 100 ms, all three dots disappeared. After a 200-ms grey blank screen, the same setup followed for the probe, including the fixation interval of 250 ms and probe onset. After the probe response, all dots disappeared and the screen turned blank for 1,200 ms, concluding one prime-probe sequence. For an example trial of a prime-probe sequence see Fig. 1. If participants responded incorrectly by looking at the wrong half of the screen, an error message appeared for 1,000 ms asking to look at the correct target.

Participants started with a practice block of 16 trials, followed by two experimental blocks each comprising 128 prime-probe sequences. The practice block consisted of four trials in which response and distractor repeated (RRDR), four trials in which response repeated, but distractor changed (RRDC), four trials in which response changed, but distractor repeated (RCDR), and four trials in which response and distractor both changed (RCDC). The experimental blocks consisted of 64 RRDR-trials, 64 RRDC-trials, 64 RCDR-trials, and 64 RCDC-trials in total. Response repetition always resembled a target location repetition, whereas a response change always resembled a target location change. Note that in response-repetition trials, there was no stimulus repetition, that is, no instruction letter repeated from prime to probe. Thereby, if distractor repetitions or changes affect response repetitions and changes, it can be deduced that these were due to distractor-*response* binding and not to distractor-*target* binding (see Giesen & Rothermund, 2014; note, however, that target repetitions in response repetitions do not necessarily lead to different distractor-response binding effects than target changes in response repetitions, Schöpper, Singh, & Frings, 2020). In response-change trials, there was no stimulus repetition as well; however, in these the letter change always indicated giving the other response. Apart from that, letter and distractor selection were set random. Every block started with a 5-point calibration and validation of the eyetracker. Participants were allowed to take a self-paced break between the two experimental blocks.

#### Results

##### Fixation screen durations

Fixation screen duration is the time between fixation dot onset and the onset of the instruction letter and distractor. This duration comprises the stable fixation of 250 ms necessary before the presentation of the instruction letter and any time that passes until said stable fixation was first established. Average fixation screen duration for the prime was 274 ms. Average fixation screen duration for the probe was 434 ms. Note that in addition to the fixation screen duration, there was a blank screen of 200 ms between prime response and probe fixation onset, as well as a blank screen of 1,200 ms between probe response and the next prime fixation onset.^2^

##### Data preparation

We analyzed latency and landing position of the first saccade in probe trials after instruction letter onset in the probe display. For meaningful data analysis a number of prerequisites were necessary. First, the probe trial had to be preceded by a valid prime trial. Hence, we had to exclude trials for which the preceding prime display ended with an error message (*N* = 533, 8.01% of all 6,656 trials). Second, a number of probe trials contained small saccades that refixated the fixation point prior to the instructed saccade to the target. Although these trials showed a successful later saccade to the target eventually, the initial re-fixation saccade might interfere with the processes we intended to study such that these trials could not be included in the analysis. For example, the intervening small saccade prolongs latency of the target saccade from probe onset. Thus, we decided to analyze only trials in which the first saccade is a saccade to the target. Hence, we excluded all saccades that landed less than 25% (i.e., 3.19°/100 px) from the fixation dot on the x-axis. These trials were quite common (*N* = 1100, 16.53% of trials). We further excluded all trials in which the fixation interval between prime and probe lasted longer than 600 ms (*N* = 320, 4.81% of trials) to control for two things: First, when distractor-response binding effects are investigated, typically an interval of 500 ms between prime response and probe target is used, yielding strong binding effects (e.g., Frings, 2011; Frings et al., 2007; Schöpper, Singh, & Frings, 2020; Singh et al., 2016); however, distractor-based retrieval is known to decline over a short period of time and is absent when using intervals of 2,000 ms (Frings, 2011). Second, a longer fixation interval might have resulted from unstable fixations, but also from additional uninstructed eye movements prior to the fixation of the fixation dot. Adding the 200-ms blank screen to our cutoff of 600 ms allows an interval of up to 800 ms between prime response completion and probe target onset. This should be short enough to observe distractor-based retrieval effects and to control for unwanted eye movements, but also long enough to avoid too much data loss caused by longer fixations. Finally, we excluded trials with saccadic latencies below 100 ms or above 1.5 interquartile range above the third quartile of a participant’s distribution (Tukey, 1977; this calculation was based on all 256 trials for each participant) (*N* = 163, 2.45% of trials). In total, our remaining data set contained *N* = 4,540 trials.

##### Vertical saccadic landing deviation

**Fig. 2:**
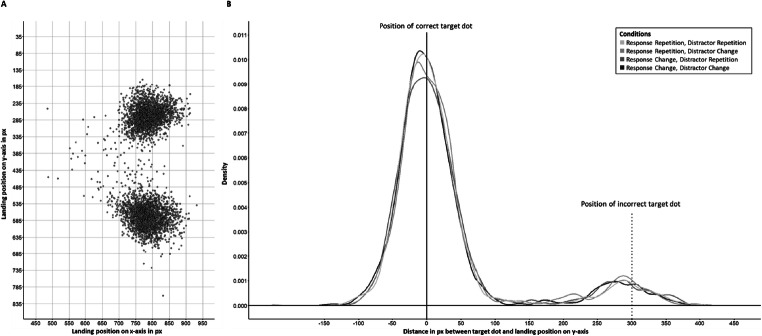
**a** Coordinates on the x- and y-axis in pixels (px) of all probe saccade landing positions of Experiment 1 after applying the exclusion criteria (see main text). Target dots were at 776 px on the x-axis and at 285 px and 585 px on the y-axis. **b** Density function of the distance in pixels between landing position on the y-axis and the target dot separate for all four conditions in Experiment 1. Negative values indicate a saccadic overshoot towards the correct half, whereas higher positive values indicate the saccade landing towards or even in the incorrect screen half. Note the small peak around 300 px visualizing saccades executed to the incorrect target dot

Figure 2a presents the saccade landing positions in the probe trials. To compare saccade accuracy between conditions we calculated the vertical saccadic landing deviation (VSLD), that is, the difference between the saccade landing position and the saccade target along the vertical axis. Data for downward targets were flipped along the horizontal such that upward and downward trials could be aligned. By this, the VSLD becomes a marker for response inaccuracy with positive/higher values indicating a stronger bias towards the incorrect screen half. Note that VSLD is not just noise around the target: A saccade can land exactly on the target on y-axis (i.e., equal to zero), can overshoot the correct target location (i.e., a negative value, indicating a bias for correct responding), or can land below the target or even on the incorrect side (i.e., a positive value). Due to these positive and negative signs of each individual landing position, the distance becomes a marker for response biases and thereby errors. Data are depicted in Fig. 2b and show the distribution of overshooting saccades (negative values) and saccades biased to or even landing on the incorrect target location (positive values). From these, the average VSLDs were calculated for all four conditions, with higher values indicating higher inaccuracy (or lower or even negative values indicating higher accuracy). For ease of interpretation, we transformed the average pixel distances into degree of visual angle.

**Fig. 3:**
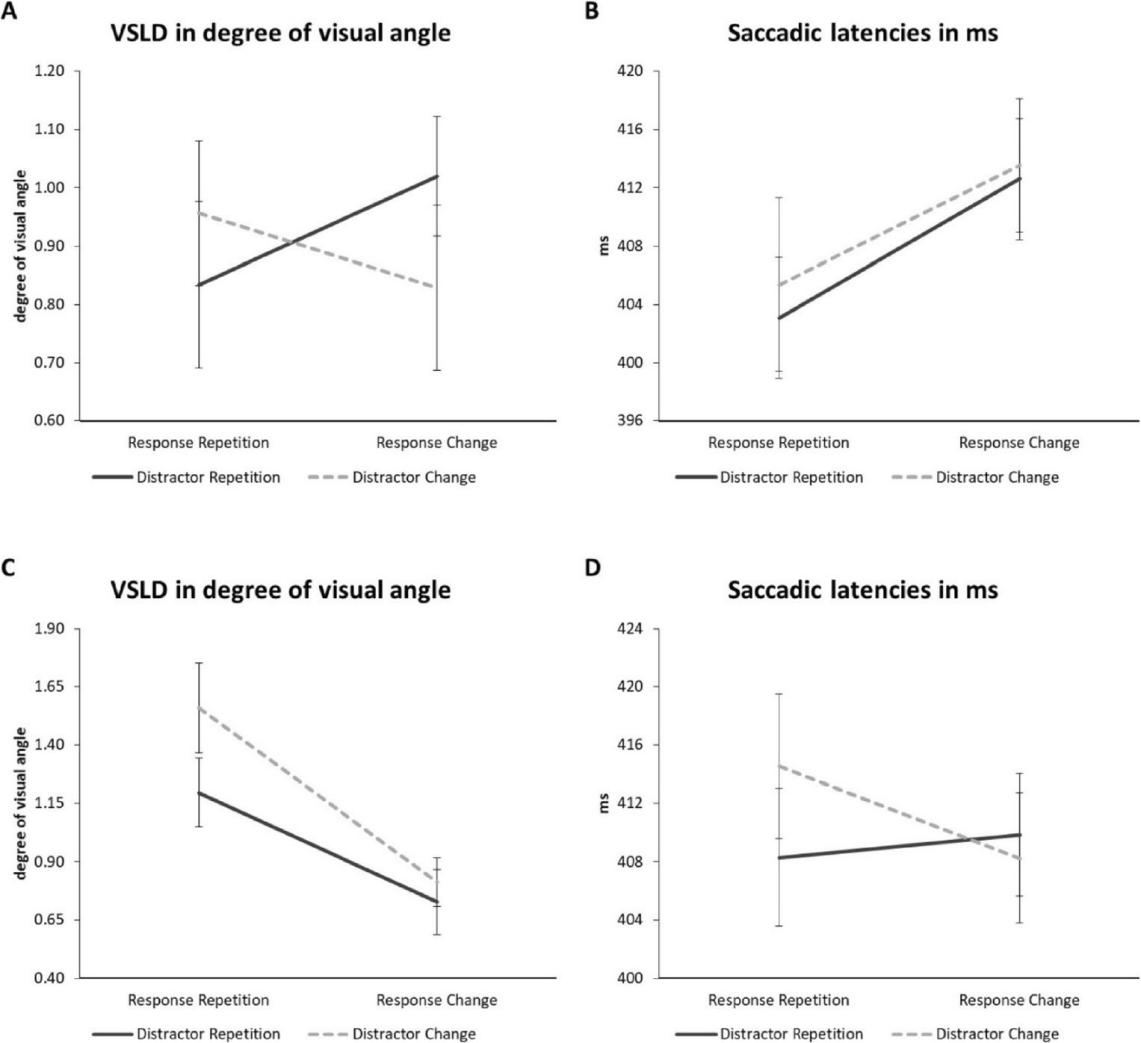
**a** Results for the average distance in degree of visual angle between target dot and the saccadic landing position on the y-axis, that is, vertical saccadic landing deviations (VSLD), of Experiment 1. **b** Results for saccadic latencies in milliseconds (ms) of Experiment 1. **c** VSLDs of Experiment 2. **d** Saccadic latencies of Experiment 2. Error bars represent within-subject standard errors after Cousineau-Morey (Cousineau, 2005; Morey, 2008)

A 2 (response relation: repeated vs. changed) × 2 (distractor relation: repeated vs. changed) repeated-measures ANOVA on VSLDs yielded no main effect of response relation, *F*(1, 25) = 0.04, *p* = .852, ${\upeta}_p^2$ < .01, and no main effect of distractor relation, *F*(1, 25) = 0.16, *p* = .695, ${\upeta}_p^2$ = .01. Crucially, there was an interaction of response relation x distractor relation, *F*(1, 25) = 5.47, *p* = .028, ${\upeta}_p^2$ = .18: When the response repeated, saccades landed closer to the target if the distractor also repeated (RRDR: distance of 26 px or 0.83°), compared to when the distractor changed (RRDC: distance of 30 px or 0.96°). When the response changed, repeating the distractor caused saccades to land further away from the target (RCDR: distance of 32 px or 1.02°) compared to when both response and distractor changed (RCDC: distance of 26 px or 0.83°). VSLDs are presented in Fig. 3a.

To highlight the presence of a binding pattern, we calculated the overall distractor-response binding effect (e.g., Frings, 2011; Schöpper, Singh, & Frings, 2020; Singh et al., 2016) by calculating a differential value of the interaction, that is, (RRDC-RRDR)-(RCDC-RCDR). The resulting value adds up the partial repetition costs for response repetitions and response changes. The binding effect for the VSLD was 0.31° (or 10 px) and significant when tested against zero in a one-sample *t*-test, *t*(25) = 2.34, *p* = .028, *d* = 0.46.

##### Saccadic latencies

Saccadic latency is the time between the onset of the target letter and the initiation of the first saccade. In addition to the cut-off criteria mentioned above, for analysis of saccadic latencies, trials were only included if the probe response was executed to the correct half of the screen and ended without an error message (comparable to the common approach of including trials in which both prime and probe responses are correct for reaction time analyses in manual response data; e.g., Singh et al., 2016; Schöpper, Hilchey, et al., 2020; Schöpper, Singh, & Frings, 2020). Due to these constraints, 38.66% of probe trials were discarded.

A 2 (response relation: repeated vs. changed) × 2 (distractor relation: repeated vs. changed) repeated-measures ANOVA on saccade latencies yielded no main effect of response relation, *F*(1, 25) = 2.27, *p* = .145, ${\upeta}_p^2$ = .08, and no main effect of distractor relation, *F*(1, 25) = 0.40, *p* = .531, ${\upeta}_p^2$ = .02. Contrary to the analysis of the VSLDs, there was no interaction of response relation x distractor relation, *F*(1, 25) = 0.05, *p* = .834, ${\upeta}_p^2$ < .01 (RRDR: 403 ms; RRDC: 405 ms; RCDR: 413 ms; RCDC: 414 ms). Saccadic latencies are presented in Fig. 3b. The overall distractor-response binding effect (RRDC-RRDR)-(RCDC-RCDR) for saccadic latencies was 1 ms and not significant when tested against zero, *t*(25) = 0.21, *p* = .834, *d* = 0.04.

#### Discussion

In Experiment 1, participants executed eye movements cued by letters that were surrounded by an irrelevant distractor-frame. VSLDs revealed that eye movements are affected by distractor-based retrieval: Participants’ initial saccades after target onset landed on average closer to the target dot, when the response as well as the distractor repeated from prime to probe. When the distractor changed, the saccade was biased towards the direction of the incorrect target dot. When the response changed, this pattern was reversed, with saccades being biased towards the incorrect target dot when the distractor repeated, but landing closer to the correct target when the distractor changed. Saccadic latencies were not affected by a binding pattern (see *General discussion*).

Event files decline after only a few seconds (Hommel & Frings, 2020) and distractor-response-binding effects are no longer observed if the interval between prime and probe is 2,000 ms (Frings, 2011; Pastötter et al., 2021). To control for this, we excluded trials from analysis if the interval between prime response and probe onset exceeded 800 ms. In turn, distractor-based retrieval should be greatly reduced if the RSI is prolonged. We conducted Experiment 2, which was identical to Experiment 1, except that the RSI was set to a longer duration. If the binding pattern is diminished here, this would be evidence for quick event file decay in eye movements comparable to manual movements (Frings, 2011; Hommel & Frings, 2020; Pastötter et al., 2021).

## Experiment 2 (eye responses; long RSI)

### Participants

Twenty-seven university students and staff of the University of Muenster (23 women, four men, *M*_*age*_ = 24.15 years, *SD*_*age*_ = 4.33, age range: 18–35) participated for course credit, a monetary reward (8 €), or voluntarily. All participants gave written informed consent and reported normal or corrected-to-normal vision.

### Apparatus, materials, design, and procedure

Experiment 2 was identical to Experiment 1, except for the following. The chin rest was positioned approximately 57 cm in front of the screen, resulting in slightly larger stimulus dimensions (fixation dot and target dots: 0.70°; Letters, length x height: 0.50° × 0.70°; Shapes: 2.01°; vertical distance between the two target dots: 10.52°). The stable fixation of the fixation dot required for the onset of the instruction letter was set to 400–650 ms. Additionally, the blank screen between prime response and probe fixation onset was 1,000 ms, and the blank screen between probe response and prime fixation onset was 2,000 ms.

#### Results

##### Fixation screen durations

Average fixation screen duration prior to the prime was 565 ms. Average fixation screen duration prior to the probe was 597 ms.

##### Data preparation

Data preparation for the trials (*N* = 6,912) of Experiment 2 was as described for Experiment 1. Trials in which the prime response was incorrect were not included in the analysis; in Experiment 2, we counted a prime response as incorrect if the landing position of the first saccade after prime target onset had been made to the incorrect screen half^3^ (*N* = 1,037, 15.00% of trials). Saccades that landed less than 25% (i.e., 3.51°/100 px) from the fixation dot on the x-axis (*N* = 882, 12.76% of trials) were excluded. We further excluded all trials in which the fixation interval between prime and probe lasted longer than 1,000 ms (*N* = 51, 0.74% of trials). Adding the 1,000-ms blank screen to our cutoff of 1,000 ms allows an interval of 1,400 and up to 2,000 ms between prime response completion and probe target onset. Finally, as with Experiment 1, we excluded trials with saccadic latencies below 100 ms or above 1.5 interquartile range above the third quartile of a participant’s distribution (Tukey, 1977) (*N* = 182, 2.63% of trials). In total, our remaining data set contained *N* = 4,760 trials.

##### Vertical saccadic landing deviation

A 2 (response relation: repeated vs. changed) × 2 (distractor relation: repeated vs. changed) repeated-measures ANOVA on VSLDs yielded a main effect of response relation, *F*(1, 26) = 16.89, *p* < .001, ${\upeta}_p^2$ = .39: Saccades landed closer to the target if the response changed (distance of 22 px or 0.77°) compared to when it repeated (distance of 39 px or 1.38°), suggesting the occurrence of IOR (e.g., Klein, 2000). The main effect of distractor relation did not reach significance, *F*(1, 26) = 3.47, *p* = .074, ${\upeta}_p^2$ = .12. The interaction of response relation x distractor relation was not significant, *F*(1, 26) = 1.33, *p* = .260, ${\upeta}_p^2$ = .05 (RRDR: distance of 34 px or 1.20°; RRDC: distance of 44 px or 1.56°; RCDR: distance of 21 px or 0.73°; RCDC: distance of 23 px or 0.81°). VSLDs are presented in Fig. 3c. The overall distractor-response binding effect (RRDC-RRDR)-(RCDC-RCDR) for VSLD was 0.28° (or 8 px) and not significant when tested against zero, *t*(26) = 1.15, *p* = .260, *d* = 0.22.

##### Saccadic latencies

For the analysis of saccade latencies, we only included trials if they met the inclusion criteria mentioned above as well as those in which the initial saccade in the probe trial landed on the correct screen half, that is, the one with the correct target. This was the case in 60.45% of trials, since there were quite a large number of VSLDs.

A 2 (response relation: repeated vs. changed) × 2 (distractor relation: repeated vs. changed) repeated-measures ANOVA on saccade latencies yielded no main effect of response relation, *F*(1, 26) = 0.21, *p* = .653, ${\upeta}_p^2$ = .01, no main effect of distractor relation, *F*(1, 26) = 0.56, *p* = .459, ${\upeta}_p^2$ = .02, and no interaction of response relation x distractor relation, *F*(1, 26) = 1.47, *p* = .236, ${\upeta}_p^2$ = .05 (RRDR: 408 ms; RRDC: 415 ms; RCDR: 410 ms; RCDC: 408 ms). Saccadic latencies are presented in Fig. 3d. The overall distractor-response binding effect (RRDC-RRDR)-(RCDC-RCDR) for saccadic latencies was 8 ms and not significant when tested against zero, *t*(26) = 1.21, *p* = .236, *d* = 0.23.

##### Between-experiment comparison

We added Experiment (Experiment 1: short RSI vs. Experiment 2: long RSI) as a between-subjects factor to the repeated-measures ANOVA, since only seven participants took part in both experiments.

##### Vertical saccadic landing deviation

A 2 (response relation: repeated vs. changed) × 2 (distractor relation: repeated vs. changed) repeated-measures ANOVA with 2 (Experiment: short RSI vs. long RSI) as a between-subjects factor on VSLDs (all statistical values correspond to viewing angle as dependent variable; pixel coordinates are presented due to completeness, however, since viewing distance differed between Experiment 1 and Experiment 2, pixel coordinates are less meaningful in their interpretation) yielded no main effect of RSI, *F*(1, 51) = 0.53, *p* = .469, ${\upeta}_p^2$ = .01. There was a main effect of response relation, *F*(1, 51) = 7.14, *p* = .010, ${\upeta}_p^2$ = .12 (RR: 34 px or 1.14°; RC: 25 px or 0.85°), that was modulated by Experiment, *F*(1, 51) = 8.69, *p* = .005, ${\upeta}_p^2$ = .15: When the RSI between prime response and probe onset was short (Experiment 1), saccadic landing positions for response repetition (28 px or 0.90°) and response change (29 px or 0.93°) trials were similar; however, when the RSI was long (Experiment 2), saccades landed closer to the target if the response changed (22 px or 0.77°) compared to when it repeated (39 px or 1.38°). The main effect of distractor relation was not significant, *F*(1, 51) = 1.58, *p* = .215, ${\upeta}_p^2$ = .03, and was not significantly modulated by RSI, *F*(1, 51) = 2.97, *p* = .091, ${\upeta}_p^2$ = .06. The overall interaction of response relation x distractor relation was significant, *F*(1, 51) = 4.50, *p* = .039, ${\upeta}_p^2$ = .08 (RRDR: 30 px or 1.02°; RRDC: 37 px or 1.26°; RCDR: 26 px or 0.87°; RCDC: 25 px or 0.82°). This interaction was not modulated by RSI, *F*(1, 51) = 0.02, *p* = .897, ${\upeta}_p^2$ = .00.

##### Saccadic latencies

The same 2 (response relation: repeated vs. changed) × 2 (distractor relation: repeated vs. changed) repeated-measures ANOVA with 2 (Experiment: short RSI vs. long RSI) as a between-subjects factor on saccadic latencies yielded no significant main effects or interactions (all F ≤ 2.04).

#### Discussion

In Experiment 2 we used the same setup as in Experiment 1 but with an RSI of 1,400–2,000 ms. In line with the quick decay of distractor-based retrieval effects (Frings, 2011), the binding effect in VSLDs was greatly diminished and no longer significant. The between-experiment comparison revealed no significant modulating role of RSI on the distractor-response binding effect. The calculated bindings effects for VSLD and latencies in Experiment 2 came with a small but existent effect size. This suggests that retrieval in Experiment 2 might not have been fully absent but operated at a much reduced level – this is consistent with recent evidence that there is interindividual variance in decay rates (Pastötter et al., 2021).

In contrast to Experiment 1, IOR drove the data pattern in VSLDs. This fits very well with the recent suggestion that retrieval masks IOR (Hilchey, Rajsic, et al., 2018) by extending it: If the RSI is short (e.g., 450–800 ms in Experiment 1), retrieval is active and masks IOR. If, however, the RSI is long (e.g., 1,400–2,000 ms in Experiment 2), so that event file retrieval is greatly reduced (Frings, 2011; Hommel & Frings, 2020), IOR – an effect being stable up to 3,000 ms (e.g., Samuel & Kat, 2003) – is no longer masked (see *General discussion*). In conclusion, eye movements can be affected by distractor-response binding processes much as manual movements. Moreover, decay of distractor-based retrieval in eye movements follows a timeline comparable to that in manual movements.

For better comparability of the effector system, we decided to conceptually replicate Experiment 1 by asking participants to manually discriminate target letters with key-press responses. Key presses are the common response type for measuring distractor-response binding effects (e.g., Frings, 2011; Frings et al., 2007; Schöpper, Singh, & Frings, 2020). If distractor-based retrieval affected the pattern in Experiment 1, using the described experimental sequence but with manual key presses as responses should lead to the observation of distractor-based retrieval – because this is the “common” way to measure this effect.

## Experiment 3 (hand control)

### Participants

Twenty-seven students of the University of Trier (15 women, 12 men, *M*_*age*_ = 24.96 years, *SD*_*age*_ = 4.50, age range: 19–36) participated for either course credit or a monetary reward (4 €). All participants gave written informed consent and reported normal or corrected-to-normal vision.

### Apparatus, materials and design

The setup was as in Experiment 1, except for the following. Participants were tested individually in dimly lit noise-isolated rooms. Experiments were conducted on screens with a display solution of 1,680 × 1,050 px and a refresh rate of 60 Hz. Due to a slightly different positioning of the chin rest at approximately 52 cm in front of the screen, all dimensions appeared slightly larger than in Experiment 1 (fixation dot and target dots: 0.77°; letters, length x height: 0.66° × 0.77°; shapes: 2.31°; horizontal distance between fixation dot and position of target dots: 14.79°; vertical distance between the two target dots: 11.20°); however, the overall dimension relations as well as actual size on the screen were approximately the same as in Experiment 1. Note that the two target dots at the right half of the screen were irrelevant for task execution and were only used to recreate the visual display used in Experiment 1.

### Procedure

The procedure was the same as in Experiment 1, except for the following. Participants were instructed to discriminate the appearing instruction letter by manually pressing the up arrow or down arrow, with their right and left index finger, respectively. The fixation screens prior to the prime and the probe varied randomly based on the average durations of visual fixations observed for Experiment 1. The fixation duration before the prime was set to randomly vary between 260 and 350 ms (Experiment 1: 274 ms). The fixation duration before a probe target was set to randomly vary between 400 and 600 ms (Experiment 1: 434 ms). This fixation interval is on average (≈ 500 ms) slightly longer than that of Experiment 1, but we wanted to allow a larger range based on the variation observed for fixation durations. In turn, the duration between prime offset and probe onset, encompassing the 200-ms blank screen and the fixation screen before probe onset, varied between 600 and 800 ms. The maximum duration of 800 ms between prime response and probe onset was the same as in Experiment 1 (where this was achieved by a cut-off). Whereas stimuli in Experiment 1 were picked randomly, we used a pseudo-random design with all letter-distractor combinations appearing equally often for each condition in the experimental trials of Experiment 3. The 16 practice trials were picked at random from the list of pseudo-random combinations.

### Error rates

Pressing the wrong key for a target was considered an error. Trials were only included for analysis if the prime response was correct. Hence, error rate is the percentage of incorrect probe responses given after a correct prime response. Due to these constraints, 3.44 % of probe trials were discarded.

**Fig. 4:**
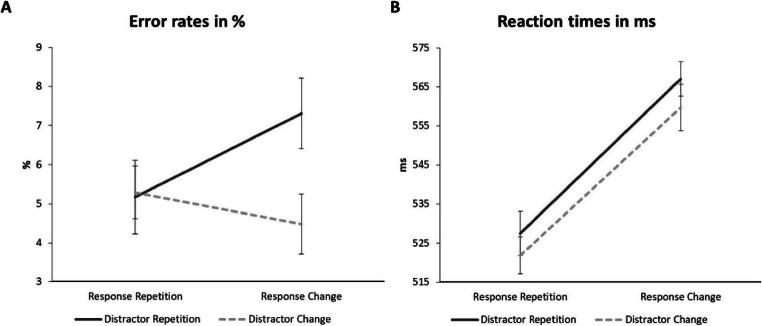
**a** Error rates in percentages and **b** reaction times in milliseconds observed for Experiment 3. Error bars represent within-subject standard error after Cousineau-Morey (Cousineau, 2005; Morey, 2008)

A 2 (response relation: repeated vs. changed) × 2 (distractor relation: repeated vs. changed) repeated-measures ANOVA on error rates gave no main effect of response relation, *F*(1, 26) = 0.46, *p* = .503, ${\upeta}_p^2$ = .02. However, the main effect of distractor relation was significant, *F*(1, 26) = 8.86, *p* = .006, ${\upeta}_p^2$ = .25. Participants made more errors in trials with distractor repetition (6.24 %) compared to distractor change (4.88 %). Crucially, there was an interaction of response relation x distractor relation, *F*(1, 26) = 5.76, *p* = .024, ${\upeta}_p^2$ = .18. For response repetitions, participants made slightly less errors if the distractor also repeated (5.17 %) compared to when the distractor changed (5.29%). For response changes on the other hand, distractor repetition caused strong interference (7.31 %) compared to a distractor change (4.48 %). Error rates are presented in Fig. 4a. The overall distractor-response binding effect for error rates (RRDC-RRDR)-(RCDC-RCDR) was 2.96% and significant when tested against zero, *t*(26) = 2.40, *p* = .024, *d* = 0.46.

### Reaction times

Reaction time is the time between onset of the target and onset of the button press. In our framework this is interpreted as comparable to the saccadic latencies, which is the time required for initiating the saccade.

Trials were only included if both prime response and probe response were correct. Only reaction times above 100 ms or below 1.5 interquartile range above the third quartile of a participant’s distribution (Tukey, 1977) were included for analysis. Due to these constraints, 12.60 % of probe trials were discarded.

A 2 (response relation: repeated vs. changed) × 2 (distractor relation: repeated vs. changed) repeated-measures ANOVA on reaction times showed a main effect of response relation, *F*(1, 26) = 31.73, *p* < .001, ${\upeta}_p^2$ = .55, with a benefit of response repetition (525 ms) over response change (563 ms). There was a main effect of distractor relation, *F*(1, 26) = 4.42, *p* = .045, ${\upeta}_p^2$ = .15, with participants reacting faster if a distractor changed (541 ms) compared to when a distractor repeated (547 ms). As with the saccadic latencies in Experiment 1, there was no interaction of response relation x distractor relation, *F*(1, 26) = 0.14, *p* = .714, ${\upeta}_p^2$ = .01 (RRDR: 527 ms; RRDC: 522 ms; RCDR: 567 ms; RCDC: 560 ms). Reaction times are presented in Fig. 4b. The overall distractor-response binding effect for reaction times, (RRDC-RRDR)-(RCDC-RCDR), was 2 ms and not significant when tested against zero, *t*(26) = 0.37, *p* = .714, *d* = 0.07.

#### Discussion

In Experiment 3 we used a design comparable to Experiment 1 but changed the effector with which a response was executed. Participants had to press a key after discriminating the instruction letter. We found the typical binding pattern in the error rates. The observed binding effect was mainly driven by response changes for which repeating the distractor caused interference, compared to when the distractor changed. However, note that there was an overall distractor change benefit, modulating the interaction. In some experimental designs, main effects – for example, overall response repetition benefits (e.g., Frings et al., 2007) or overall distractor repetition benefits (e.g., Experiment 2 in Schöpper, Singh, & Frings, 2020) – additionally modulate distractor-response binding effects. While it is possible that some (additional) processes or components in responding are to some degree effector-specific (cf., Bompas et al., 2017), our goal was to simply observe distractor-based retrieval. Congruent with our hypothesis, this pattern occurred in Experiment 1 and Experiment 3, that is, in saccadic and manual response accuracy. As with the saccadic latencies in Experiment 1, there was no distractor-response binding effect in reaction times of Experiment 3.

##### Reaction time distribution analysis

**Fig. 5:**
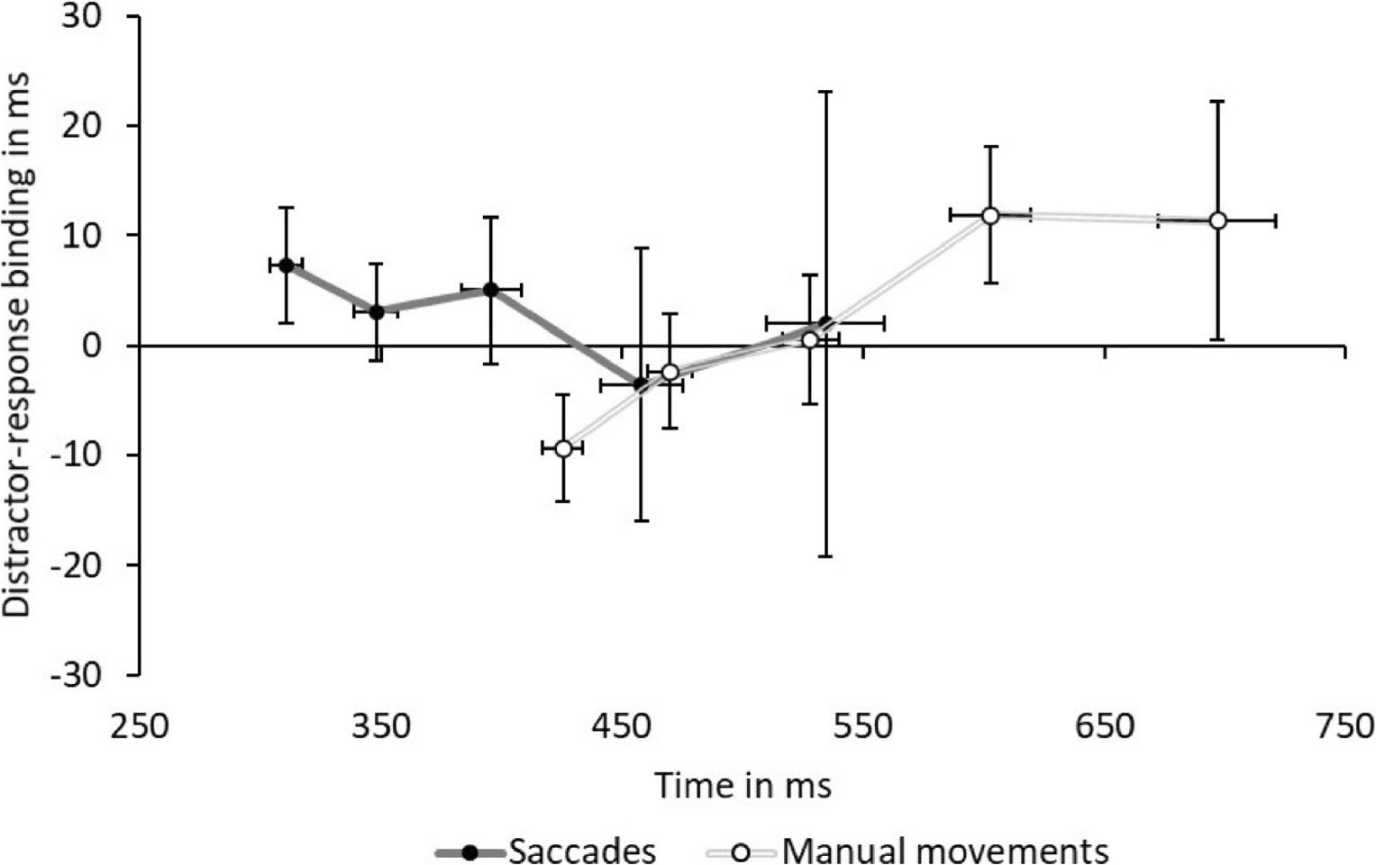
The calculated interaction of response relation and distractor relation, that is, the distractor-response binding effect, on the y-axis and reaction times on the x-axis as a function of percentile (cf. delta plots; see De Jong et al., 1994; Ridderinkhof, 2002), separate for Experiment 1/saccadic latencies (dark grey line) and Experiment 3/manual reaction times (light grey striped line). The black (saccades) and white (manual movements) dots represent the 10th, 25th, 50th, 75th, and 90th percentiles for each function. See main text for explanations. Error bars represent standard error of each mean of each averaged percentile for the differential value (y-axis) and overall reaction times (x-axis)

In both Experiment 1 (saccades) and Experiment 3 (manual movements) we observed distractor-based retrieval in accuracy-related variables, but not in saccadic latencies or manual reaction times. Retrieval has been found to take time to emerge, that is, if the response is executed too fast, retrieval has no chance to affect it (“horserace-account”; Frings & Moeller, 2012; Schöpper, Hilchey, et al., 2020; cf. Neill, 1997). If so, retrieval could have affected late responses in our experiments (e.g., Chao & Hsiao, 2021; Schöpper & Frings, 2022). To test for this, we took the 10th, 25th, 50th, 75th, and 90th percentiles for every condition and each participant (cf. Schöpper & Frings, 2022; Taylor & Ivanoff, 2005), and calculated the interactions as distractor-response binding effects separate for each percentile. Next, we calculated a MANOVA with the factor of percentile (10th vs. 25th vs. 50th vs. 75th vs. 90th) on the calculated binding effects separately for each experiment. In the saccadic task, the main effect of percentile^4^ was not significant, *F*(4, 22) = 0.88, *p* = .490, ${\upeta}_p^2$ = .14 (10th percentile: 7 ms; 25th percentile: 3 ms; 50th percentile: 5 ms; 75th percentile: -4 ms; 90th percentile: 2 ms). In contrast, in the manual task, the main effect of percentile was significant, *F*(4, 23) = 3.81, *p* = .016, ${\upeta}_p^2$ = .40 (10th percentile: -9 ms; 25th percentile: -2 ms; 50th percentile: 1 ms; 75th percentile: 12 ms; 90th percentile: 11 ms). This suggests that for saccades, retrieval did not occur at late responses; however, it did so at late manual responses. Lastly, we plotted the calculated effects for each percentile against the average response speed of the respective percentile (cf. delta plots; De Jong et al., 1994; Ridderinkhof, 2002). In Fig. 5 it becomes visible that slowest saccadic latencies were in the range of intermediate manual reaction times, suggesting that saccadic latencies might have simply been too fast for late-emerging distractor-based retrieval.

## General discussion

We investigated whether eye movements are affected by distractor-based retrieval. For this matter, participants had to discriminate target letters surrounded by irrelevant shapes and execute their response by looking at one of two target positions in Experiment 1. VSLDs revealed a distractor-response binding-effect: Saccades landed closer to the target dot when the response and distractor fully repeated or fully changed, compared to when they partially repeated. In line with quick event file decay in manual movements (Frings, 2011; Hommel & Frings, 2020), this binding pattern was greatly reduced and not significant in Experiment 2 in which the RSI was long. Finally, in Experiment 3 – in which participants performed the same task as in Experiment 1 but executed their response with a manual movement – the same binding effect in error rates was observed.

Additionally, saccadic latencies in Experiment 1 and reaction times in Experiment 3 were both unaffected by distractor-based retrieval. Reaction time distributions (cf. Schöpper & Frings, 2022; Taylor & Ivanoff, 2005) revealed that this might have been caused by overall fast responding in that distractor-based retrieval might affect saccadic latencies if more time had been given (cf. Frings & Moeller, 2012). While in many experiments binding effects emerge both in reaction times and error rates (e.g., Singh et al., 2016; discrimination tasks in Schöpper, Hilchey, et al., 2020), some studies found binding patterns only emerging in one dependent measure (e.g., only in reaction times; Frings & Rothermund, 2017; Frings et al., 2007; Singh & Frings, 2020). Thus, it is possible that some configurations of our display (e.g., letters, distractor shapes, distances, etc.), our experimental design (e.g., only response repetitions without target repetitions; in turn, introducing response repetitions with target repetitions might increase retrieval; see also Giesen & Rothermund, 2014), task demands, or other (unknown) components have led to the absence of a binding pattern emerging in response times for both effectors. Alternatively, the letter discrimination task could have been executed so fast that retrieval had no chance to affect the speed of responding (cf. Frings & Moeller, 2012). Yet, more importantly, the same task but executed with different effectors resulted in comparable binding patterns in response accuracy.

Response accuracy in Experiment 3, the manual task, was measured as the percentage of incorrect probe responses after correct prime responses, which is the common way to assess error rates in the distractor-response binding paradigm (e.g., Schöpper, Singh, & Frings, 2020). Response accuracy in Experiment 1 and Experiment 2, the saccadic task, was operationalized as the deviation away from the target on the y-axis, with larger negative values indicating an overshoot above the correct target dot and larger positive values indicating more deviation towards the incorrect target dot. All conditions resulted in positive deviation values. We interpret this deviation as being caused by saccades deviating towards the incorrect target dot, in the most extreme as a response executed to the wrong location. Note that we assume this deviation to only occur on the y-axis: As we varied responses to be executed to two target dots only differing in their location on the y-axis, any systematic bias should only affect this vertical dimension. By this, response accuracy measured as VSLD becomes a continuous measurement compared to, for example, a discrete measurement of manual key presses. To summarize, while both dependent variables do not measure exactly the same thing, they both depict a measure for response accuracy.

In compound search tasks in visual search (e.g., Töllner et al., 2008) – a type of discrimination task in which partial repetition costs as in binding experiments are observed (for a discussion, see Frings et al., 2020; Schöpper, Hilchey, et al., 2020) – a serial processing of the target until response generation has been proposed (e.g., Zehetleitner et al., 2012): First the target is identified, followed by the selection of a response, which ultimately leads to response execution. Zehetleitner et al. (2012) identified the second stage as being crucial for observing partial repetition costs (see also Töllner et al., 2012). Thus, it can be argued that tasks lacking the component of a post-selection stage prior to responding do not lead to binding effects, and that a translation of a target feature into the associated response (i.e., by applying a specific stimulus-response mapping; Henson et al., 2014) is necessary for observing this (cf., Töllner et al., 2012; Zehetleitner et al., 2012). Accordingly, when an eye movement simply occurs to localize or orient to a target (Hilchey, Rajsic, et al., 2018), that is, the response directly follows target identification, no binding effects are observed. However, if the target has to be post-selectively processed in that the target feature has to be translated into the associated response (current study; e.g., “P” ➔ “look up”), eye movements are affected by binding processes. This explanation also fits well with binding being absent in simple visual detection and localization performance, but present in discrimination performance (Huffman et al., 2018; Schöpper, Hilchey, et al., 2020), when executed with manual responses.

In our experiments we compared two tasks that differed in the effector executing the response. Of course, each experimental design has its peculiarities due to the effector involved. For example, in the saccadic discrimination task, participants made use of the whole display, whereas participants in the manual discrimination task could always remain on the location of the fixation dot and instruction letter. Moreover, we qualitatively compared the pattern in saccadic landing positions with the pattern of error rates in the manual task, with both obviously being differently calculated dependent variables. However, we think that experiments involving different modalities (e.g., Spence, Lloyd, McGlone, Nicholls, & Driver, 2000) or different effectors (e.g., Bompas et al., 2017) always necessarily differ due to such effector specificities. Thus, we propose that both experiments were designed to be as comparable as possible for what we intended to investigate with further differences being unavoidable due to the effectors involved.

With this mindset, the steps in identifying the target and selecting a response based on a letter being presented at the fixation point were the same for both our saccadic and manual discrimination tasks. Hence, only the response execution, that is, the motor system of the effector, differed between experiments. In turn, potentially all cognitive processes that have led to the binding effects in our experiments should be comparable until response execution, where effector-specific differences might apply (like timing; see also, e.g., Bompas et al., 2017). While the current study supports effector-invariant processing of integration and retrieval, further research, potentially involving neurophysiological measurements, is needed to gain more evidence. For example, it would be fascinating to see how responses of single neurons in crucial areas for oculomotor performance such as the superior colliculus, the lateral intraparietal area, or the frontal and supplementary eye fields depend on a trial-by-trial basis on performance (and neural firing patterns) in preceding trials with similar or different targets. Further, we used key presses as responses for manual movements; whereas the experiment fulfilled its purpose by showing a pattern of distractor-based retrieval, future studies might look at similarities or potential effector differences arising in manual response executions more comparable to saccades, like manual pointing movements (cf., Baldauf et al., 2006; Deubel & Schneider, 2003; Paprotta et al., 1999).

Our results fit well with previous studies showing saccades affecting subsequent response executions, or saccades being affected by previously perceived information or prior response executions. Pratt and Abrams (1999) conducted a number of discrimination tasks using cue-target designs in which the authors varied the repetition or change of cue/target identity and cue/target locations and varying saccadic and manual responses (or giving no responses). If no eye movement to the cue was made (i.e., no response was given), IOR was observed for the subsequent manual response to the target. Crucially, if an eye movement was made to the cue, repeating the cue identity as the target identity led to the disappearance of IOR. The authors suggested that overt attention produced a repetition priming effect. Given the current study, *responding* with an eye movement to the cue as part of a discrimination performance led to the integration of stimulus and response features – which were subsequently retrieved by the manual response to the target (in accordance with rather abstract event file representation; Moeller et al., 2015). Pfeuffer et al. (2016) described that participants performed (uninstructed) saccades to locations at which they expected an effect of a previous manual response. The authors interpret such saccades as serving to monitor manual action outcomes. Van der Stigchel and Theeuwes (2006) found that saccades deviated away from the location where a distractor was previously presented (see also Belopolsky & Van der Stigchel, 2013). Talcott and Gaspelin (2020) found a location priming effect for saccades in several search tasks. Initial saccades were biased towards a previous location. This location priming effect for eye movements was strongest for targets with repeating features, which the authors explain by (pre-attentional) episodic retrieval processes in visual search (e.g., Huang et al., 2004). Ultimately, benefits for feature repetitions when performing eye movements have been found in several visual search tasks (e.g., Becker & Horstmann, 2009; Kruijne & Meeter, 2016; McPeek et al., 1999; Shurygina et al., 2019).

The present results show that distractor-based retrieval mechanisms underlying actions executed with eye movements are comparable to the mechanisms underlying manual responses. Although the neurophysiological processing differs at some point (see *Introduction*), the performance outcome in a discrimination task is similar, suggesting effector-invariant processing of integration and retrieval (or at least similar underlying principles leading to this; Bompas et al., 2017; Logan & Irwin, 2000). One has to bear in mind, however, that such retrieval-based mechanisms for eye movements occurred in a situation requiring a discriminatory judgment at fixation (the letter, in our case). In naturalistic situations (e.g., Hayhoe & Ballard, 2005; Liversedge & Findlay, 2000), this is typically not the case. Instead, eye movements are executed towards visual objects that are to be discriminated in the periphery. In this case, the discrimination follows after the eye movement. Such a situation (e.g., Hilchey, Rajsic, et al., 2018) does not show binding effects. Yet, our study shows that this is not because distractor-based retrieval does not exist for eye movements – in fact, it can influence gaze behavior as it does for manual movements, given specific situational demands. Rather, whether or not saccades are influenced by retrieval in naturalistic environments depends on those situational demands.

The experimental setup of using two positions in the periphery – one being the target location, the other being a distractor location – often leads to the observation of saccades landing intermediate between both locations, that is, the global effect (for a review see, e.g., Van der Stigchel & Nijboer, 2011). However, in our experiment, the saccade was not triggered by a peripheral onset but by an endogenous instruction letter. Additionally, as can be seen in Fig. 2a, saccades landed in roughly symmetrical circles at the two target locations and only a few intermediate; thus, the pattern of VSLDs resembled erroneous responses and/or biases due to the respective trial conditions and was not caused by a general bias to an intermediate location due to a global effect. Finally, the global effect typically affects fast saccades (e.g., Ottes, Van Gisbergen, & Eggermont, 1985) and declines with increasing saccadic latency (e.g., Coëffé & O’Regan, 1987; Heeman, Theeuwes, & Van der Stigchel, 2014). The relatively long saccadic latencies in our experiments are unlikely to be affected by the global effect. Further, such a constant bias associated with eye movements should equally affect all experimental conditions and therefore cannot account for the differences we observed. Yet, the current results might spur on new research on the global effect or other eye-movement biases being affected by partial repetition effects and/or the duration of RSIs.

We only observed IOR in our eye-movement data when the RSI was long (Experiment 2), not when it was short (Experiment 1). As already mentioned, this can be attributed to task demands (Huffman et al., 2018; Schöpper, Hilchey, et al., 2020), and binding effects masking any potentially active IOR processes (Hilchey, Rajsic, et al., 2018). Whereas this explanation also allows us to explain the absence of IOR in manual responses (Experiment 3), the complete lack of IOR in the eye-movement data of Experiment 1 can be seen as more puzzling. Saccadic IOR (for a review see, e.g., Klein & Hilchey, 2011) is attributed to attentional (or sensory; Satel & Wang, 2012; Wang et al., 2012) and oculomotor mechanisms (Kingstone & Pratt, 1999; Taylor & Klein, 2000) and is seen as a result of prior oculomotor responding (Hilchey, Rajsic, et al., 2018). Yet, we did not see any sign of prior oculomotor activation causing IOR in Experiment 1. In our saccadic discrimination task, participants responded to *both* targets with a saccade; thus, after executing a saccade to the target dot in the prime display, the fixation dot appeared in the periphery, causing sensory IOR mechanisms (as in Taylor & Klein, 2000; see Wang et al., 2012, for a discussion), and oculomotor IOR for the fixation dot (see Wang et al., 2011). This is followed by the succeeding probe display demanding the next saccadic response.^5^ Thus, a saccade executed to *either* of both target dots on the right half of the screen would have suffered to some extent from the previous saccade orienting to the fixation dot on the left half of the screen, as a consequence of oculomotor IOR (Wang et al., 2011, 2012).

Alternatively, it is also possible that saccadic responses were indeed affected by IOR through prior oculomotor activation, but that this was balanced out by binding or retrieval processes. In the manual reaction times of Experiment 3, we observed a benefit for repeating the response, which is a common finding in manual discrimination tasks (e.g., Frings et al., 2007; Schöpper, Hilchey, et al., 2020). In VSLDs and latencies of Experiment 1, we neither found a benefit for repeating nor for changing the response. Thus, it is possible that retrieval processes (i.e., a benefit for repeating the response) balanced out IOR processes (i.e., a benefit for changing the response; see also Experiment 3, saccade task, in Pratt & Abrams, 1999). However, this post hoc explanation is speculative and based on a null effect. Thus, further research is necessary when (oculomotor) IOR in eye movements is not observed – as in location priming (Talcott & Gaspelin, 2020), some experimental designs involving anti-saccades (e.g., Eng et al., 2017), endogenous cueing (Li & Lin, 2002), or the current study (Experiment 1) – and whether it is completely absent, diminished, or, given the possibility offered by the current results, masked by retrieval processes.

## Conclusion

Eye movements as well as manual movements are both affected by distractor-based retrieval. Our results confirm the tacitly assumed effector invariance in action control theories (e.g., Frings et al., 2020; Hommel, 2004; Prinz, 1998) for responses executed with eye movements.

## Footnotes

1 Additionally, targets in Hilchey, Rajsic, et al. (2018) could be very small (“invisible condition“) compared to regular sized (“visible condition“), with the idea that small targets would definitely require an eye movement to them to give the manual discrimination response. In Experiment 2, the targets could also appear in the same or a different color. Although these factors had some influence on performance, the overall interpretation remains the same.

2 The different durations for fixations preceding the prime and fixations preceding the probe might be explained by the time participants have available after giving a response to move their gaze back to the area the fixation dot appeared in, as well as IOR towards the fixation dot (e.g., Wang et al., 2011; see *General discussion*).

3 Due to a difference in programming, displayed error messages in Experiment 2 were not logged as in Experiment 1. However, results of Experiment 1 remained stable by applying the criterion reported here to said data.

4 Percentile also modulated the factor of response relation in Experiment 1. Due to this, we calculated the main effect as a differential value, that is, ((RCDR+RCDC)/2)-((RRDR+RRDC)/2). A positive value indicates a response repetition benefit, whereas a negative value indicates a response change benefit. Percentile modulated this effect, *F*(4, 22) = 6.13, *p* = .002, ${\upeta}_p^2$ = .53, in that for the 10th (22 ms), the 25th (21 ms), and the 50th (14 ms) percentiles there was a response repetition benefit, which disappeared during the 75th (0 ms) percentile and turned into a response change benefit at the 90th (-18 ms) percentile. This might be interpreted as late-emerging IOR (compare Schöpper & Frings, 2022; Taylor & Ivanoff, 2005), but also hints at a possible interplay of retrieval and IOR (see *General discussion*).

5 Note that instruction letters always appeared at fixation; thus, saccades were always executed based on endogenous target processing and never due to orienting to targets in the periphery (compare, e.g., Hilchey, Rajsic, et al., 2018). Following Wang et al. (2012), we would not expect IOR based on sensory, but on oculomotor mechanisms, at least for prime and probe responses.
